# The present state of the leech fauna (Annelida, Hirudinea) in the Upper Irtysh cascade of water reservoirs

**DOI:** 10.3897/zookeys.596.7886

**Published:** 2016-06-07

**Authors:** Lyudmila I. Fedorova, Irina A. Kaygorodova

**Affiliations:** 1 Irkutsk State Agrarian University named after A.A. Ezhevsky, Timiryazev Street, 59, 664038 Irkutsk, Russia; 2 Limnological Institute, Siberian Branch of Russian Academy of Sciences, Ulan-Batorskaya Street, 3, 664033 Irkutsk; Russia; 3 Irkutsk State University, Sukhe Bator Street, 5, 664003, Irkutsk, Russia

**Keywords:** Hirudinea, species diversity, Upper Irtysh water reservoirs

## Abstract

Hirudinea is a small and ecologically important group of aquatic organisms. However, up to date, the leech fauna of Kazakhstan is poorly studied. The presence of large under-collected areas, such as the Upper Irtysh basin, makes biodiversity studies concerning these invertebrates from Kazakhstan relevant. In this paper, the latest information on species diversity of the freshwater hirudofauna of the Upper Irtysh cascade of water reservoirs, the Kazakhstan part of Irtysh River, is presented. It includes 10 free-living and parasitic species, of which 7 and 9 inhabit the Shulbinsk and the Bukhtarma reservoirs, respectively. These species belong to 2 orders, 3 families and 6 genera. The faunal list highlights four potentially new morphological species (*Alboglossiphonia* sp., *Erpobdella* sp., *Piscicola* sp. 1 and *Piscicola* sp. 2). Besides them, another three species *Erpobdella vilnensis*, *Helobdella stagnalis* and *Theromyzon tessulatum* recorded for the first time in the area. The exact systematic position is stated for all leech taxa. Each species from the list accompanied with information on taxonomic synonymy, data on its geographic distribution, and brief summary of morphological and ecological characteristics.

## Introduction

The Irtysh River with a catchment area of more than 300 000 km^2^ is the main water artery of the Eastern Kazakhstan, which meets the regional requirements of water resources. The hydrological level of the river is controlled by the Upper Irtysh water cascade consisting of Bukhtarma, Shulbinsk and Ust-Kamenogorsk storage reservoirs. Using these reservoirs, a multi-year, seasonal and weekly regulation of the river flow is consistently implemented (Beysembaeva and Dubrovskaya 2014, Vinokurov et al. 2010). The Bukhtarma reservoir is the largest of them; it serves as the main regulator of the cascade as a whole. The close proximity of the mining resource industry and nonferrous metallurgy resulted in ecologically strenuous conditions on the Bukhtarma reservoir (Kulikov et al. 2011). A specificity of biota inhabiting the Bukhtarma reservoir was formed by both the natural dispersal of species from the flooded water bodies and by the use of artificial colonization (Zadoenko et al. 1985, Evseeva 2011). The Ust-Kamenogorsk reservoir is located in the mountain valley of canyon type; it regulates weekly and daily river flow. This reservoir is characterized by significant water exchange, cold water and absence of the littoral zone. A high flowage of the reservoir, with an extremely unstable water exchange and low water temperature are limiting factors for many species of aquatic organisms. The aquatic fauna of this reservoir was formed as a result of biological invasions from upstream reservoirs (Evseeva 2012). The Shulbinsk reservoir in turn completes the cascade of artificial reservoirs, constructed in the Upper Irtysh. The reservoir conducts the seasonal adjustment of lateral afflux (Ulba River and Uba River) in the area between Bukhtarma and Shulbinsk hydropower stations. Operating mode of the Shulbinsk reservoir has a negative impact on its ichthyofauna and causes a significant reduction in biodiversity of benthic organisms (Kulikov et al. 2011). Thus, the exploitation of hydroelectric power stations, increased water consumption and the development of floodplain areas have led to changes in the hydrological regime of the Irtysh River and decrease in the natural potential of the river ecosystem (Beysembaeva and Dubrovskaya 2014). Instability of the hydrological level in the Irtysh cascade reservoirs, industrial impacts, and acclimatization activities conducted earlier necessitate the studying of natural biodiversity and its preserving.

Freshwater Hirudinea is one of the most important ecological groups of hydrobionts. Leeches are of scientific interest as an important link in the food chain of aquatic ecosystems, as well as bio-indicators of water pollution (Bezmaternykh 2007, Romanova and Klimina 2010, Kaygorodova et al. 2014). Moreover, parasitic leeches are involved in the abundance regulation of host species. As it was established, Hirudinea sp. may be directly related to transmission of bacterial and viral infections (Ahne 1985, Mulcahy et al. 1990, Cruz-Lacierda et al. 2000, Faisal et al. 2009, Faisal et al. 2011), as well as hematozoa including trematodes, cestodes, and nematodes (Demshin 1975) and parasitic flagellates (Khan 1976, Khamnueva and Pronin 2004, Burreson 2007), which are considered to be pathogenic organisms for aquatic animals. Moreover, ulceration, hemorrhage, and inflammation associated with leech attachment sites weaken the host and may pose an opportunity hosts to bacterial infections.

Previously, special-purpose research of leech fauna has never been conducted within the Upper Irtysh cascade. There are only scant data on the occurrence of a few leech species in the Bukhtarma water reservoir. As part of the fish parasite fauna, two species of leeches were identified – *Piscicola geometra* (Linnaeus, 1758) and *Caspiobdella fadejewi* (Epstein, 1961) (Izumova 1977). Later on, the presence of *Piscicola geometra* has been confirmed, and the species list has been supplemented by *Hemiclepsis marginata* (Müller, 1774) and *Erpobdella octoculata* (Linnaeus, 1758) (Deviatkov 2012). The presence of *Caspiobdella fadejewi* is doubtful, since this species belongs to the European faunistic complex and has a limited distribution within the basins of the rivers that flow into the Azov and Black Seas (Epstein 1987) and the Volga River (Lapkina and Komov 1983). Data on its occurrence outside this region are absent. Most likely, the leech species affiliation could be incorrectly identified. Thus, the species composition of the Irtysh leech fauna needs to be clarified, especially since the previously mentioned papers exclude the possibility of a verification due to the absence of morphological descriptions.

The aim of the study was to determine the current state of the species diversity and its spatial distribution and to fulfil a comparative analysis of the parasitic annelid fauna of the Upper Irtysh water cascade.

## Materials and methods

**Figure 1.**
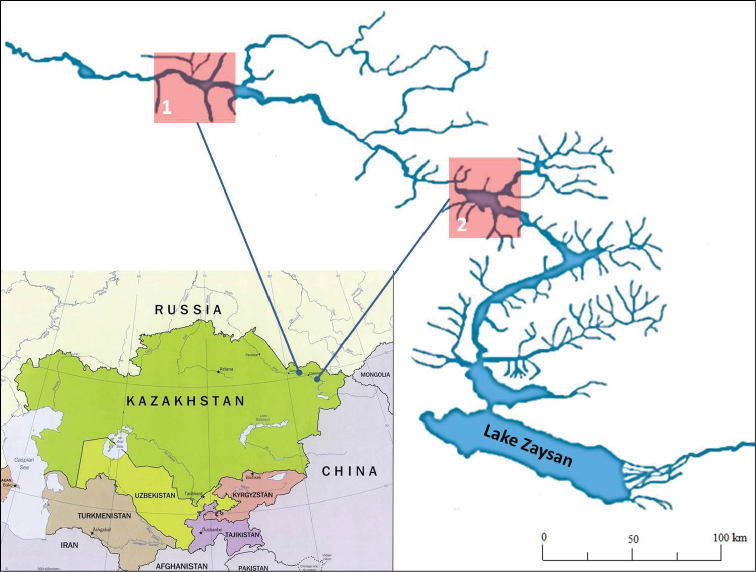
Geographical location of the study region with indication of sampling areas: **1** Shulbinsk water reservoir **2** Bukhtarma water reservoir.

Expedition works were conducted in August-September 2014. The biological material was collected in the dam areas of Bukhtarma and Shulbinsk water storage reservoirs (Fig. 1). Catching leeches was performed manually or by using hydrobiological nets in the coastal zone of each reservoir in a depth range of 0.5–1.5 m. The specimens were fixated in 80% ethanol with preliminary animal anaesthesia by low percent alcohol solution.

Morphological analysis of ethanol-fixed samples was performed using a stereomicroscope MSP-2 var. 2 (LOMO). The leech species affiliation was ascertained under existing systematic keys (Lukin 1976, Nesemann and Neubert 1999) in accordance with the present-day classification of the group. Reference specimens are stored in the annelid collection of the Limnological Institute.

## Results

This is the updated checklist of the Irtysh leech species inhabiting the Upper Irtysh cascade of water reservoirs. At present, 10 species were documented. The species diversity includes leeches from two orders (Rhynchobdellida and Arhynchobdellida), three families (Glossiphoniidae, Piscicolidae, Erpobdellidae) and six genera (*Alboglossiphonia*, *Helobdella*, *Hemiclepsis*, *Theromyzon*, *Piscicola*, and *Erpobdella*). Species composition includes both free-living and parasitic freshwater leeches. Parasitic forms are represented by seven species, and include representatives of 5 genera – *Theromyzon tessulatum*, *Hemiclepsis marginata*, *Alboglossiphonia* sp., *Helobdella stagnalis*, *Piscicola geometra*, *Piscicola* sp. 1, *Piscicola* sp. 2. Among free-living macrophagous leeches, there are only three erpobdellid species, namely *Erpobdella vilnensis*, *Erpobdella octoculata*, and *Erpobdella* sp.

The taxonomic list includes both Palaearctic species (*Hemiclepsis marginata*, *Piscicola geometra*, *Theromyzon tessulatum*, *Erpobdella octoculata*, *Erpobdella vilnensis*), and widespread Holarctic species (*Helobdella stagnalis*). Four species in the checklist, including *Alboglossiphonia* sp., *Erpodella* sp. and two representatives of *Piscicola* (Table 1), were referred in this paper, with caution, to unidentified species since their morphology differed from all currently described species. With high probability, these four unidentified species are potentially new to science. All four of these, for the first time were recorded within the Irtysh River basin.

**Table 1.** Leech fauna of the Upper Irtysh water cascade

#	Taxon	Bukhtarma reservoir	Shulbinsk reservoir
1.	*Alboglossiphonia* sp.	+	+
2.	*Helobdella stagnalis*	+	+
3.	*Hemiclepsis marginata*	+*	+
4.	*Theromyzon tessulatum*	+	
5.	*Piscicola geometra*	+	+
6.	*Piscicola* sp. 1		+
7.	*Piscicola* sp. 2	+	
8.	*Erpobdella octoculata*	+	+
9.	*Erpobdella vilnensis*	+	+
10.	*Erpobdella* sp.	+	
**Total**:		**9**	**7**

* according to Deviatkov (2012).

Hirudofauna of the Bukhtarma and Shulbinsk water reservoirs is represented by 9 and 7 leech species, respectively (Table 1). Higher species diversity found in the Bukhtarma storage reservoir, is probably related to more favourable environmental conditions for freshwater leeches. This reservoir has a greater area and more tributaries (Fig. 1). Moreover, 50 years ago exactly in the Bukhtarma reservoir, experiments on acclimation of fish, molluscs, mysids and amphipods have been conducted. Sixteen alien species (6 species of fish and 10 species of invertebrates) have successfully taken root (Zadoenko et al. 1985, Evseeva 2011), which significantly expanded the number of potential hosts for parasitic leeches.

## Systematics

### Phylum: ANNELIDA Lamarck, 1809 Class: CLITELLATA Michaelsen, 1919 Subclass: HIRUDINEA Lamarck, 1818 (synonym Hirudinida)

#### Order: RHYNCHOBDELLIDA Blanchard, 1894

##### Family: GLOSSIPHONIIdae Vaillant, 1890

###### Subfamily: Glossiphoniinae Autrum, 1939

####### *Alboglossiphonia* Lukin, 1976

**Geographic distribution.** Holarctic.

**Type species.** *Alboglossiphonia heteroclita* (Linnaeus, 1761)

####### *Alboglossiphonia* sp.

Fig. 2

**Figure 2.**
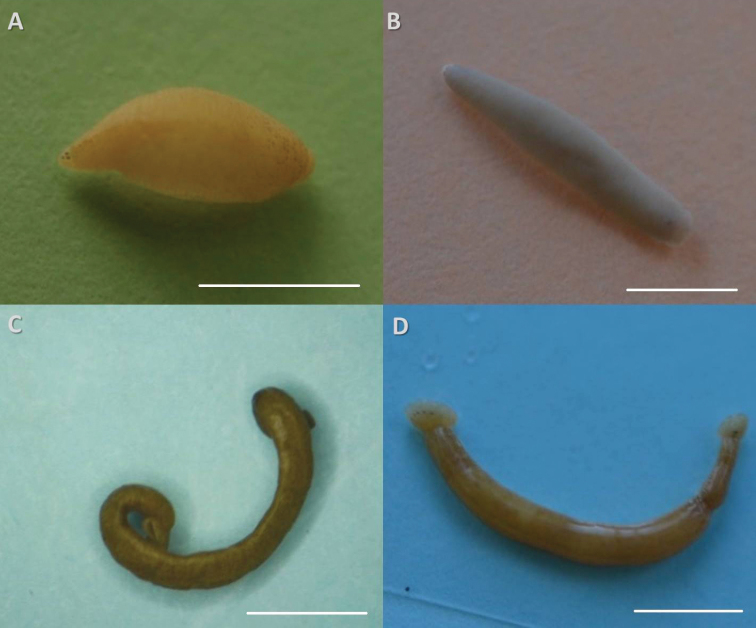
External morphology of new species from the Upper Irtysh reservoirs: **A** *Alboglossiphonia* sp. **B** *Erpobdella* sp. **C** *Piscicola* sp. 1 **D** *Piscicola* sp. 2. Scale bar is 5 mm.

**New species records.** The Shulbinsk and the Bukhtarma water reservoirs.

**Morphological characteristics.** Small leaf-shaped leech. Mature individuals are about 3–6 mm in length and 2–4 mm in width. The number and arrangement of eyes is typical for representatives of the genus. Body surface is smooth, papillae are mild. Live animals are light yellow, the colour practically disappears when fixing. A characteristic feature of this species is a specific arrangement of the sparse pigment cells, main concentration of which falls on the anterior and posterior end of the body surface. *Alboglossiphonia* sp. is bears offspring on the ventral surface; dimensions of young are up to 1 mm in length with a width of 0.2 mm.

**Ecological characteristics.** This species is a benthic ectoparasite of snails. It parasitizes mainly gastropods. Single specimens were found often on aquatic vegetation, less often on solid substrates.

####### *Helobdella* Blanchard, 1876

**Geographic distribution.** Cosmopolite.

**Type species.** *Helobdella stagnalis* (L., 1758)

####### *Helobdella stagnalis* (Linnaeus, 1758)

**Synonymy.** *Hirudo stagnalis*: Linnaeus 1758; *Hirudo pulligera*: Daudin 1800; *Glossiphonia perate*: Johnson 1816; *Erpobdella bioculata*: Lamark 1818; *Clepsine bioculata*: Savigny 1822; *Glossiphonia punctata* Johnson 1825; *Clepsine stagnalis*: Fillipi 1837; *Glossiphonia bioculata*: Maquin-Tandon 1846; *Glossiphonia circulans*: Maquin-Tandon 1846; *Clepsine modesta*: Verrill 1972; *Glossiphonia modesta*: Vaillant 1890; *Glossiphonia stagnalis*: Blanchard 1894; Glossiphonia (Helobdella) stagnalis: Moore 1922; *Bakedebdella gibbosa*: Sciacchitiano 1939.

**Geographic distribution.** Cosmopolite. Shulbinsk and Bukhtarma water reservoirs.

**Morphological characteristics.** One pair of eyes. Leeches are oval in shape; body has jagged edges. The body surface is smooth, no papillae. Intravital coloration can be from light green to yellowish or even with tints of gray. Preserved specimens become dirty-white or completely lose colouring. A distinctive feature of this species is the presence of chitin plate on the dorsal side. Small leeches, reaching a body length of 2–11 mm and width of 1–4 mm.

**Ecological characteristics.** This species is considered one of the most common freshwater leeches in the world. It inhabits almost all types of freshwaters, the most abundant in slow running and stagnant waters with well-developed aquatic vegetation. This species is a benthic ectoparasite. It sucks the hemolymph of freshwater invertebrates such as oligochaetes, larvae of insects, and molluscs.

####### *Hemiclepsis* Vejdowsky, 1884

**Geographic distribution.** Palaearctic region.

**Type species.** *Hemiclepsis marginata* (Müller, 1774)

####### *Hemiclepsis marginata* (Müller, 1774)

**Synonymy.** *Hirudo marginata*: Müller 1774; *Hirudo variegata*: Braun 1805; *Hirudo cephalota* Carena 1820; *Hirudo oscillatoria*: Saint-Amas 1825; *Piscicola tesselata*: Maquin-Tandon 1826; *Piscicola linearis*: Kollar 1842; *Glossobdella cephalota*: Blainville 1827; *Haemoharis marginata*: Filippi 1837; *Glossiphonia marginata*: Maquin-Tandon 1846; *Hirido flava*: Dalyell 1853; *Glossiphonia flava*: Johnston 1865; *Glossiphonia marginata*: Blanchard 1892.

**Geographic distribution.** Palaearctic region. Shulbinsk water reservoir.

**Morphological characteristics.** Two pairs of eyes, the first pair is a smaller and located close to the front edge of the body. Clavate head is well separated from the body. Colour in life is from light green to light brown. Alcohol-fixed specimens are either greyish green or light pink colour, respectively. The dorsal side is granular and pigmented, with the exception of the posterior part and suckers with a characteristic pattern. Small leeches with length of 2–12 mm and 2–6 mm of width.

**Ecological characteristics.** This species is found in differing types of running and stagnant waters. This leech is an ectoparasite sucking blood of different species of fish, amphibians and birds.

####### *Theromyzon* Philippi, 1867

**Geographic distribution.** Palaearctic region.

**Type species.** *Theromyzon tessulataum* (Müller, 1774)

####### *Theromyzon tessulatum* (Müller, 1774)

**Geographic distribution.** Palaearctic region. Bukhtarma water reservoir.

**Synonymy.** *Hirudo tessulata*: Müller 1774; *Nephelis tessulatum*: Savigny 1822; Erpodella vulgaris var. tessulatum: Blainville 1828; *Clepsine tessulata*: Müller 1844; *Glossiphonia tessulatum*: Moquin-Tandon 1846; *Glossiphonia vitrina*: Johnston 1865; *Theromyzon tessulatum*: Philippi 1867; *Hemiclepsis tessulatum*: Vejdovsky 1884; *Protoclepsis tessulatum*: Livanow 1902.

**Morphological characteristics.** Four pairs of eyes. The shape and colour of the body varies and depends on satiety of leeches. Colour in life is from light green to brown-red. Posterior sucker is well developed, with radial bright spots on it. The dorsal surface is almost smooth, with six rows of paramedial underdeveloped papillae on it. The crop has 7 pairs of caeca. Leeches are up to 15 mm in length and 10 mm in width.

**Ecological characteristics**. It was found in warmed backwaters with well-developed aquatic vegetation. *Theromyzon tessulatum* is a temporary ectoparasite feeding on vertebrates, mostly waterfowl.

##### PISCICOLIDAE Johnston, 1865 (synonym Ichthyobdellidae Leuckart, 1863)

###### *Piscicola* De Blainville, 1818

**Geographic distribution.** Holarctic region

**Type species.** *Piscicola geometra* (Linnaeus, 1758)

###### *Piscicola geometra* (Linnaeus, 1758)

**Geographic distribution.** Holarctic region. Both the Shulbinsk and the Bukhtarma water reservoirs.

**Synonymy.** *Hirudo geometra*: Linnaeus 1758; *Hirudo galearia*: Braun 1805; *Ichthyobdella geometra*: Blainville 1828; *Ichthyobdella percae*: Templeton 1836; *Piscicola piscium*: Apáthy 1888; *Piscicola lippa*: Olsson 1893; *Piscicola perspicax*: Olssen 1893.

**Morphological characteristics.** Two pairs of eyes are typical for the family. Elongated small sized cylindrical leeches with well-developed (conspicuous) suckers. Cranial sucker is smaller than caudal one. There are 10 ocelli on caudal sucker. Body colour varied from greenish to brown-olive. A characteristic feature of the species is cross-shaped pattern on the dorsal surface. Leeches are 5–12 mm of length and 1.5–2 mm of width.

**Ecological characteristics**. This species inhabits both the Bukhtarma and Shulbinsk water reservoirs. Ectoparasite of various fish species.

###### *Piscicola* sp. 1

Fig. 2

**New species record.** the Bukhtarma reservoir and the Shulbinsk reservoir.

**Morphological characteristics.** Number and arrangement of the eyes is typical for Piscicolidae. Suckers are small. Posterior sucker with diameter of 1.5 mm is slightly larger than anterior sucker. There is an achromatous medial stripe on the dorsal surface. Ethanol fixed specimen has a light green coloration, with dark pigment cells, which are evenly distributed over the entire body surface. Body length of 17 mm and a width of 2 mm

**Ecological characteristics.** A single specimen was found in a floating piece of rotten wood in the coastal zone of the Shulbinsk reservoir. Host is unknown.

###### *Piscicola* sp. 2

Fig. 2

**New species record.** the Bukhtarma water reservoir.

**Morphological characteristics.** Two pairs of eyes, their arrangement as in *Piscicola geometra*. The body is in the form of an elongated cylinder. It has a light brown coloring. Distinguishing features are rather large copulatory area and diamond-shaped depigmented pattern on the dorsal side. In fixed specimens, body length of 12–16 mm and a width of 1.5–2.5 mm.

**Ecological characteristics.** Three specimens were collected in swampy backwater of the Bukhtarma reservoir near the New Bukhtarma village in the water plant thickets, where many invertebrates and young fish live. Exact host is unknown.

#### Order: ARHYNCHOBDELLIDA Blanchard, 1894

##### Suborder: Erpobdelliformes Sawyer, 1986

###### Family: ERPOBDELLIDAE Blanchard, 1894

####### *Erpobdella* de Blainville, 1818

**Geographic distribution.** Holarctic region

**Type species.** *Erpobdella octoculata* (Linnaeus, 1758)

####### *Erpobdella octoculata* (Linnaeus, 1758)

**Synonymy.** *Hirudo octoculata*: Linnaeus 1758; *Hirudo vulgaris*: Müller 1774; *Nephelis octoculata*: Moquin-Tandon 1826; *Nephelis sexoculata*: Scheider 1883; *Nephelis scripturata*: Scheider 1885; *Nephelis atomaria*: Blanchard 1893; *Nephelis crassipunctata*: Scheider 1893; *Nephelis seripturata*: Blanchard 1893; *Nephelis sexoculata*: Blanchard 1893; *Herpobdella octoculata*: Johannson 1910; *Herpobdella octomaculata*: Pawlowski 1935.

**Geographic distribution.** Palaearctic region. Shulbinsk and Bukhtarma water reservoirs.

**Morphological characteristics.** Four pair of eyes. The basic colour of live specimens is light brown, with dark reticulate pigmentation in dorsal side. Body surface is covered with multiple papillae of various size. Genital pores are separated by 2.5 annuli. Leeches are up to 32 mm in length and 4 mm in width.

**Ecological characteristics.** Eurythermal species is very tolerant of a lack of oxygen and can live in highly polluted waters (Lukin, 1976). Within the studied area, *Erpobdella octoculata* is widespread, but not abundant in the Bukharma water reservoir. This macrophagous leech feeds on small invertebrates, remains of dead animals, and with the critical shortage of natural food can be cannibals.

####### *Erpobdella vilnensis* (Liskiewicz, 1925)

**Synonymy.** Herpobdella atomaria var. monostriata: Gedrouć 1916; Herpobdella octoculata subsp. vilnensis: Liskiewicz 1925; Herpobdella octoculata f. monostriata: Gedroyć and Pawlowski 1936; *Herpobdella vilnensis*: Liskiewicz 1934; Erpobdella octoculata f. monostriata: Gedroyć and Pawlowski 1937; *Erpobdella monostriata*: Gedroyć and Pawlowski 1948.

**Geographic distribution.** Palaearctic region. *Erpobdella vilnensis* is rather a common leech species that occurs in Central, Eastern and South-Eastern Europe. In Central Asia, it was first discovered in the Shulbinsk and the Bukhtarma water reservoirs during this study.

**Morphological characteristics.** Four pairs of eyes. In living leeches, the basic body is dark brown to almost black. Dorsally, there is one pair of dark paramedian longitudinal stripes. After fixation colour is changed to light brown. Genital pores are separated by 2.5 annuli. Body length is up to 22 mm at width of 3–4 mm.

**Ecological characteristics**. Predator of small invertebrates. The specimens were collected in coastal part of the Bukhtarma and Shulbinsk reservoirs. In the latter, it is a very common leech.

####### *Erpobdella* sp.

Fig. 2

**New species record.** The Bukhtarma water reservoir.

**Morphological characteristics.** Four pair of eyes with typical for the family location. Small-sized erpobdellids, with body length of 5–15 mm and width of 2–2.5 mm. The leeches have no pigmentation. Genital pores are separated by 2.5 annuli.

**Ecological characteristics.** This species was found in Northern part of the Bukhtarma reservoir only, where it very abundant.

## References

[B1] Ahne W (1985) *Argulus foliaceus* L. and *Piscicola geometra* L. as mechanical vectors of spring viraemia of carp virus (SVCV). Journal of Fish Diseases 8: 241–242. doi: 10.1111/j.1365-2761.1985.tb01220.x

[B2] Beysembaeva MA Dubrovskaya LI (2014) Estimation of water runoff perennial change of the Upper Irtysh River for water resource stability. Tomsk State University Journal 379: 189–195.

[B3] Bezmaternykh DM (2007) Zoobentos as an indicator of water ecosystems state in Western Siberia. Ecology. A series of analytical reviews of world literature 85: 1–86.

[B4] Burreson EM (2007) Hemoflagellates of Oregon marine fishes with the description of new species of Trypanosoma and Trypanoplasma. Journal of Parasitology 93(6): 1442–1451. doi: 10.1645/GE-1220.1 18314692 10.1645/GE-1220.1

[B5] Cruz-Lacierda ER Toledo JD Tan-Fermin JD Burreson EM (2000) Marine leech (*Zeylanicobdella arugamensis*) infestation in cultured orange-spotter grouper *Epinephelus coioides*. Aquaculture 185: 191–196. doi: 10.1016/S0044-8486(99)00356-7

[B6] Demshin NI (1975) Oligochaeta and Hirudinea as intermediate hosts of helminthes. Nauka, Novosibirsk, 190 pp.

[B7] Deviatkov VI (2012) On diversity of Bukhtarma reservoir macrozoobenthos in 2005–2009. Bulletin of Kazakh National University. Ecology 1(33): 162–165.

[B8] Epstein VM (1987) Annelida. In: Skarlato OA (Ed.) Key of the Freshwater Fish Parasites of the USSR Fauna, Vol. 3(2). Nauka, Leningrad, 340–372.

[B9] Evseeva AA (2011) Peculiarities of ripus for using of nutritive base in the Bukhtarma storage reservoir. Bulletin of Kazakh National University. Biology 5(51): 56–61

[B10] Evseeva AA (2012) Zooplankton of Ust-Kamenogorsk water reservoir. Bulletin of Kazakh National University. Ecology 1(33): 165–168.

[B11] Faisal M Schulz C Eissa A Whelan G (2011) High prevalence of buccal ulcerations in largemouth bass, *Micropterus salmoides* (Centrarchidae) from Michigan inland lakes associated with Myzobdella lugubris Leidy 1851 (Annelida: Hirudinea). Parasite 18(1): 79–84. doi: 10.1051/parasite/2011181079 21395209 10.1051/parasite/2011181079 PMC3671407

[B12] Faisal M Schulz CA (2009) Detection of Viral Hemorrhagic Septicemia virus (VHSV) from the leech *Myzobdella lugubris* Leidy, 1851. Parasite Vectors 282(1): . doi: 10.1186/1756-3305-2-45 10.1186/1756-3305-2-45 PMC2761889 19785752

[B13] Izumova NA (1977) Parasitofauna of fishes in reservoirs of the USSR and the way of its formation. Nauka, Leningrad, 284 pp.

[B14] Kaygorodova IA Mandzyak NB Petryaeva EY Pronin NM (2014) Genetic diversity of leeches in Lake Gusinoe (Eastern Siberia, Russia). The Scientific World Journal, 1–11. doi: 10.1155/2014/619127 10.1155/2014/619127 PMC4270114 25544958

[B15] Khamnueva TR Pronin NM (2004) New Kinetoplastid Species (Kinetoplastida: Kinetoplastidea). In: Timoshkin OA (Ed.) Index of Animal Species in Inhabiting Lake Baikal and its Area 1(2). Nauka, Novosibirsk, 1255–1260.

[B16] Khan RA (1976) The life cycle of *Trypansoma murmanensis* Nikitin. Canadian Journal of Zoology 54: 1840–1949. doi: 10.1139/z76-214 991015 10.1139/z76-214

[B17] Kulikov EV Kirichenko OI Kulikova EV Deviatkov VI Evseeva AA (2011) Recommendations to improve the status of fisheries waters of the Irtysh-Zaisan Basin. Astana, 46 pp.

[B18] Lapkina LN Komov VT (1983) New data on the finding of the leech *Caspiobdella fadejewi* in water reservoirs of the Volga. Parazitologiya 17(1): 79–97.

[B19] Lukin EI (1976) Leeches of fresh and saline waters (fauna of the USSR. Leeches). Nauka, Leningrad, 484 pp.

[B20] Mulcahy D Klaybor D Batts WN (1990) Isolation of infectious hematopoietic necrosis virus from a leech (*Piscicola salmositica*) and a copepod (Salminocola sp.), ectoparasites of sockeye salmon *Oncorhynchus nerka*. Diseases of Aquatic Organisms 8: 29–34. doi: 10.3354/dao008029

[B21] Nesemann H Neubert E (1999) Clitellata, Branchiobdellada, Acanthobdellada, Hirudinea. In: Schwoebel J Zwig P (Eds) Susswasserfauna von Mitteleuropa. Spectrum Akademischer Verlag, Heidelberg, Berlin 6(2): 1–178.

[B22] Romanova EM Klimina OM (2010) Bioresources class Hirudinea in the area of the middle Volga region: ecological significance and prospects. Proceedings of the Samara Scientific Center of the Russian Academy of Sciences 1(12): 208–211.

[B23] Vinokurov YuI Chibilev AA Krasnoyarova BA Pavleychik VM Platonova SG Sivokhip ZhT (2010) Regional Ecological Problems in Transboundary Basins of the Urals and Irtysh Rivers. Izvestiya Rossiiskoi Akademii Nauk, Seriya Geograficheskaya 3: 95–104.

[B24] Zadoenko IN Leis OA Grigoriev VF (1985) Results and Perspectives of the Baikal gammarids acclimation in reservoirs of the USSR. Collected scientific papers of the State Research Institute of Lake and River management 232: 30–34.

